# AGE DIFFERENCES IN AFFECT DYNAMICS: APPLYING A FORMALIZED THEORETICAL APPROACH

**DOI:** 10.1093/geroni/igad104.0980

**Published:** 2023-12-21

**Authors:** Maria Wirth, Andreas Voss, Klaus Rothermund

**Affiliations:** Friedrich Schiller University Jena, Jena, Thuringen, Germany; University Heidelberg, Heidelberg, Baden-Wurttemberg, Germany; Friedrich Schiller University Jena, Jena, Thuringen, Germany

## Abstract

Emotional aging research is dominated by the idea of age-related improvements that result from shifts in motivation. Socioemotional selectivity theory (SST) proposes that as individuals age, they increasingly favor emotion-related goals and savor positive but avoid negative emotions. Previous age-comparative studies on everyday emotional experience typically were descriptive or studied the processes underlying emotional experience in isolation. We aimed at a more holistic approach to test hypotheses derived from SST regarding age-related differences in general emotional dispositions (i.e., anchoring), emotional reactivity, and emotion regulation by using a comprehensive computational approach. We applied our Model of Intraindividual Variability in Affect (MIVA) to data on everyday emotional experiences in an age-diverse sample (N = 378, age range 14 – 86 years). Parameter estimations were carried out within a Bayesian framework. Our results provide partial support for predictions derived from SST. Consistent with SST, affect elicited by pleasant events was down-regulated less by older adults and affect elicited by unpleasant events was down-regulated more by older compared to younger adults. Inconsistent with SST, anchoring showed a negative age-related trend, indicating a more positive general affect disposition in younger, not older adults. Reactions to pleasant events showed no age-related differences. Reactivity to unpleasant events was highest in midlife and lower for younger and older adults. We discuss the broader implications of our approach for understanding emotional development across the lifespan.

